# Effects of the intravenous administration of purine nucleosides guanosine or inosine against hemorrhagic shock in pigs

**DOI:** 10.1186/cc10802

**Published:** 2012-03-20

**Authors:** A Schmidt, D Otsuki, DO Souza, J Auler

**Affiliations:** 1 Faculdade de Medicina da Universidade de São Paulo, Brazil

## Introduction

Hemorrhagic shock leads to the appearance of substances in plasma that depress Na/K ATPase activity, an effect that could be related to significant morbidity and mortality. Recently, some findings indicated that purine nucleosides such as guanosine, inosine or adenosine might prolong survival in shocked rats, an effect potentially related to the stimulation of Na/K ATPase activity. This study aimed to evaluate the effects of intravenous administration of guanosine or inosine combined with lactate Ringer solution (LR) on hemodynamic and oxygenation parameters and survival in an experimental model of hemorrhagic shock (HS).

## Methods

HS was induced in 24 pigs (25 to 30 kg) by blood removal for 20 minutes to target a mean arterial pressure (MAP) of 40 mmHg, which was maintained for 60 minutes with additional blood removal or retransfusion. Animals were treated with LR alone (three times the volume of blood withdrawn) or associated to 1 mmol/l guanosine or 1 mmol/l inosine. Hemodynamic and oxygenation parameters were evaluated at baseline, after HS, immediately after fluid resuscitation, and 30, 60, 120, 240 and 360 minutes after fluid resuscitation. Primary outcome was post-shock survival. Statistical analysis of parametric data was performed with one-way ANOVA for repeated measures followed by Student-Newman-Keuls. Kruskal-Wallis followed by the Dunn test was used for analysis of nonparametric data. The post-shock survival was evaluated by the Kaplan-Meier curve.

## Results

The hemodynamic and oxygenation parameters were not significantly different among pigs treated with RL alone or in combination with guanosine or inosine. No effects on post-shock survival were observed in any group.

## Conclusion

The actual preliminary results did not demonstrate any additional improvement induced by guanosine or inosine on the hemodynamic and oxygenation parameters or on the post-shock survival during HS. These findings need to be confirmed in a larger group of animals and further investigation with cellular and biochemical analysis may help to elucidate the effects of guanosine and inosine during HS.
